# Identification of QTLs Associated With Agronomic Traits in Tobacco *via* a Biparental Population and an Eight-Way MAGIC Population

**DOI:** 10.3389/fpls.2022.878267

**Published:** 2022-06-06

**Authors:** Yutong Liu, Guangdi Yuan, Huan Si, Ying Sun, Zipeng Jiang, Dan Liu, Caihong Jiang, Xuhao Pan, Jun Yang, Zhaopeng Luo, Jianfeng Zhang, Min Ren, Yi Pan, Kefan Sun, He Meng, Liuying Wen, Zhiliang Xiao, Quanfu Feng, Aiguo Yang, Lirui Cheng

**Affiliations:** ^1^Key Laboratory of Tobacco Improvement and Biotechnology, Tobacco Research Institute, Chinese Academy of Agricultural Sciences, Qingdao, China; ^2^Zhengzhou Tobacco Research Institute, China Tobacco Gene Research Centre, Zhengzhou, China; ^3^School of Agriculture, Yunnan University, Kunming, China

**Keywords:** agronomic traits, QTL, MAGIC population, RIL population, tobacco

## Abstract

Agronomic traits such as plant height (PH), leaf number (LN), leaf length (LL), and leaf width (LW), which are closely related to yield and quality, are important in tobacco (*Nicotiana tabacum* L.). To identify quantitative trait loci (QTLs) associated with agronomic traits in tobacco, 209 recombinant inbred lines (RILs) and 537 multiparent advanced generation intercross (MAGIC) lines were developed. The biparental RIL and MAGIC lines were genotyped using a 430 K single-nucleotide polymorphism (SNP) chip assay, and their agronomic traits were repeatedly evaluated under different conditions. A total of 43 QTLs associated with agronomic traits were identified through a combination of linkage mapping (LM) and association mapping (AM) methods. Among these 43 QTLs, three major QTLs, namely *qPH13-3, qPH17-1*, and *qLW20-1*, were repeatedly identified by the use of various genetically diverse populations across different environments. The candidate genes for these major QTLs were subsequently predicted. Validation and utilization of the major QTL *qLW20-1* for the improvement of LW in tobacco were investigated. These results could be applied to molecular marker-assisted selection (MAS) for breeding important agronomic traits in tobacco.

## Introduction

Tobacco (*Nicotiana tabacum* L.) is an economically important species, and it is widely planted worldwide (Xiang et al., 2016). Tobacco leaves are made into various kinds of tobacco products for human consumption. Leaf-related traits such as plant height (PH), leaf number (LN), leaf length (LL), and leaf width (LW) determine not only tobacco yield but also the quality and marketability of tobacco products (White et al., 1979; Xu et al., 2000). Improvements to these important agronomic traits are the primary targets of tobacco breeders.

These important agronomic traits are considered typical quantitative traits regulated by multiple genes, and they are affected by numerous environmental factors (Xu et al., 2000; Xiao et al., 2006). To improve these traits, the use of quantitative trait locus (QTL) mapping together with molecular marker-assisted selection (MAS) is a good strategy (Mohan et al., 1997; Wang and Guo, 2010). The development of molecular markers that are tightly linked to these important traits greatly increase the efficiency of breeding ideal cultivars. For a long time, QTL mapping and the development of markers in tobacco were mainly performed on the basis of simple sequence repeat (SSR) markers (Gholizadeh et al., 2012; Tong et al., 2016). Several QTL mapping studies of tobacco have been reported, including studies on resistance to diseases (e.g., black shank, brown spot, and powdery mildew) (Stavely et al., 1984; Shah et al., 2018; Sun et al., 2018; Zhang et al., 2018) as well as to the yield and quality of tobacco (e.g., agronomic traits and chemical components) (Julio et al., 2006; Vijay et al., 2010; Tan et al., 2012; Cheng et al., 2015). However, to date, only a few studies involving QTL mapping of important agronomic traits of tobacco have been reported. Using SSR markers based on a recombinant inbred line (RIL) population, Tong et al. (2012) performed QTL mapping of seven agronomic traits. A total of three dynamic QTLs related to the number of leaves and leaf area were identified during the three developmental stages by the F_2_ population (Song et al., 2019).

Traditional genetically different populations for QTL mapping are constructed via two parents exhibiting different target traits and include F_2_, backcross (BC), doubled haploid (DH), and RIL populations. However, these populations based on biparental populations have several limitations. First, only two alleles between two parents can be compared, while the diversity of target trait alleles with the germplasm is ignored. Second, because recombination is based on F_1_ meiosis, and most progenies harbor large fragment recombination, the accuracy of QTL detection is limited (Kearsey and Farquhar, 1998).

Compared with biparental populations, multiparent advanced generation intercross (MAGIC) populations offer significant advantages in terms of analyzing multiple alleles and providing increased recombination and mapping resolution (Cavanagh et al., 2008; Holland, 2015). To date, MAGIC populations have been widely used in QTL mapping in crop species (Bandillo et al., 2013; Li et al., 2013; Meng et al., 2016; Bossa-Castro et al., 2018; Ogawa et al., 2018). The first application of the MAGIC population on plants was reported in 2009 for *Arabidopsis thaliana* when a QTL associated with flowering time was detected using a 19-way MAGIC population (Kover et al., 2009). In rice, different types of MAGIC populations were constructed to assess QTLs for important traits such as biotic/abiotic stress, yield, and quality traits (Bandillo et al., 2013; Meng et al., 2016). In Solanaceae crop species, two eight-way MAGIC populations were constructed, and QTLs related to agronomic traits and resistance to biotic stress were identified (Pascual et al., 2014; Campanelli et al., 2019). However, the construction and utilization of MAGIC populations in tobacco have not been reported.

In this study, the biparental population and the first eight-way MAGIC population of tobacco were produced. The QTLs associated with PH, LN, LL, and LW were identified by the use of the biparental RIL population and the eight-way MAGIC population across different environments. Using a BC population and 211 tobacco accessions, we further verified the genomic location of the major QTL. These results add to the knowledge concerning the genetic basis of different agronomic traits and offer an opportunity to improve target traits via MAS in tobacco.

## Materials and Methods

### Plant Material

The cigar line Beinhart1000-1 (hereafter referred to as BH) from the USA and the flue-cured tobacco variety Xiaohuangjin1025 (hereafter referred to as XHJ) from China were used as parental lines for a biparental population. Two hundred and nine F_8_ RILs were developed by the single-seed descent method. Eight different types of tobacco accessions were used to construct an eight-way MAGIC population. BH and Florida301 are cigar-type tobacco varieties. Vam is a burley type tobacco. Basma and Samsun are oriental-type tobacco varieties. Xiaohuaqing (hereafter referred to as XHQ) and Tangpeng (hereafter referred to as TP) are sun-cured tobacco varieties from China. Honghuadajinyuan (hereafter referred to as HD) is a flue-cured tobacco variety. The eight parents were crossed pairwise to produce four two-way hybrids, and these four two-way hybrids were intercrossed in pairs to obtain six four-way crosses. One hundred and thirty-two eight-way crosses were developed by intercrossing between the six 4-way crosses. The progeny derived from the eight-way crosses was mated at random for two generations. In each generation, a minimum of 200 crosses were made between 400 different individuals. Furthermore, three individual progenies per eight-way cross were maintained. Finally, more than eight hundred eight-way MAGIC homozygous lines were generated by the single-seed descent method.

### Phenotyping

The two parents and 209 biparental RILs were planted in three different environments in accordance with a randomized complete block design with two replications. A single trial was carried out at the Xichang Experimental Station, Tobacco Research Institute, Chinese Academy of Agricultural Sciences, Xichang (27.8°N, 101.5°E, 1,500.0m elevation), Sichuan, China in 2017; the other trials were conducted at the Zhucheng Experimental Station, Tobacco Research Institute, Chinese Academy of Agricultural Sciences, Zhucheng (36.4°N, 119.1°E, 19.3m elevation), Shandong, China in 2017 and 2018. The eight parents and 537 MAGIC lines were planted in Zhucheng (36.4°N, 119.1°E, 19.3m elevation), Shandong, China, in 2019 and Longshan (28.5°N, 109.3°E, 600.2m elevation), Hunan, China in 2019 and 2020 in accordance with a randomized complete block design with two replications. In all the trials, one replication of each line consisted of two 10-plants rows with a row length of 10m, a row space of 1.2m, and a plant distance of 0.5 m.

At the flowering stage, five plants in the middle of each plot were randomly sampled for phenotypic evaluation, and the average value of five plants per plot was used for analysis. Four important agronomic traits were measured, including PH (calculated as the height of the stem from the soil surface to stem apex), LN, LL, and LW. Pearson correlation coefficients between the traits were calculated using R/4.05 software. The analysis of variance (ANOVA) functionality in QTL IciMapping version 4.1 (Meng et al., 2015) was used for ANOVA on the phenotypic traits and estimation of heritability.

### Genotyping and Construction of a High-Density Genetic Linkage map

At the Zhengzhou Tobacco Research institute, a 430 K tobacco single-nucleotide polymorphism (SNP) array composed of 432,362 markers was used to genotype the different materials (which included nine parents, 209 RIL, and 537 MAGIC lines) at the Zhengzhou Tobacco Research Institute. The SNP genotyping procedure was performed as described by Zhang et al. (2017). The genotypes for one SSR marker (PT30174) were identified using the PCR method according to the results reported by Bindler et al. (2011). To construct a high-density SNP genetic linkage map, SNPs exhibiting polymorphism between BH and XHJ were selected. SNP markers showing significant segregation distortion (*X*^2^ test, *P* < 0.01, df = 1), distortion with poor quality, or distortion with more than 10% of missing data were excluded from the map construction. IciMapping 4.1 software was used to group and order the markers. The nnTwoOpt algorithm was used to determine the preliminary order and positions of linked markers. The Rippling algorithm (with a window size of five markers) was used to adjust the linkage map. The Kosambi centimorgan function was ultimately used to calculate map distances.

### QTL Analysis

For linkage mapping (LM), inclusive composite interval mapping was implemented using IciMapping 4.1 software for QTL mapping. An empirical logarithm of odds (LOD) threshold of 3.40 was used for declaring significant QTLs based on 1,000 runs and a type I error of 5% (Meng et al., 2015). The genetic maps and QTLs were drawn using MapChart 2.0 software (Voorrips, 2002).

For association mapping (AM), a quality check was carried out according to several standards: First, the genetic positions of the markers were clearly mapped onto linkage groups (LG) *via* LM. Second, all the markers with a minor allele frequency of <5% were removed. Finally, SNP markers with more than 10% missing data were excluded. A total of 3,282 SNP markers were selected for the genome-wide association study (GWAS). The genotypic data from those markers and the phenotypic data across the three environments were analyzed *via* a mixed linear model (MLM) analysis by TASSEL 5.0 software (Bradbury et al., 2007). According to the adjusted Bonferroni method, a *p*-value of 1.52 × 10^−5^ (*P* = 0.05/Ne, Ne = 3,282) was determined to be the threshold for declaring a significant association (Li C. et al., 2019).

### Expression Levels of Candidate Genes *via* RNA-seq Analysis

To analyze the expression levels of candidate genes, plants of tobacco varieties, BH and XHJ, were grown in 12 cm-diameter plastic pots in an artificial climate chamber with a 12/12 h light/dark photoperiod at 25°C with 70–85% relative humidity. There were three repetitions, each with five plants in one replication. Leaves of the same five plants from each replication were collected for RNA-seq analysis during the flowering stage. Total RNA was isolated from the leaves using the TRIzol reagent (Life Technologies, USA) according to the manufacturer's instructions. Transcriptome sequencing was carried out by Novogene (Beijing, China) on an Illumina NovaSeq platform using the 150 bp paired-end read mode. The expression quantity was calculated as fragments per kilobase of exon per million fragments.

### Resequencing of the Different Parental Lines

To compare the difference in coding sequence regions of candidate genes, we randomly broke the genomic DNA of 9 parental lines (XHJ, BH, Florida301, Vam, Basma, Samsun, TP, XHQ, and HD) at 350 bp and used them to construct library using the TruSeq Library Construction Kit. The constructed library was sequenced on the Illumina platform by Novogene (Beijing, China), and 150 bp paired-end reads were generated. The raw sequencing data were filtered using Trimmomatic. After data filtering, we used the clean data for subsequent analyses. A total of 20-fold mean genome coverage for each sample was achieved. Furthermore, SNPs in the encoding sequence regions of the candidate genes between different parental lines were analyzed based on the resequencing results.

### Validation and Utilization of the Major QTL

A total of 211 tobacco accessions were collected and genotyped based on the marker bin20-185. The phenotypic values were measured at the Zhucheng (Shandong Province) and Xichang (Sichuan Province) sites between 2014 and 2015. The same designs as previously mentioned were used.

One hundred thirty-three BC_4_F_3_ introgression lines (ILs) derived from the flue-cured tobacco K326 (recurrent parent, GG genotype) and the oriental tobacco Samsun (donor parent, AA genotype) were generated. The phenotype of each BC_4_F_3_ line was measured at the Xishuangbanna Experimental Station, Xishuangbanna (22.1°N, 101.3°E, 1000.8m elevation), Yunnan, China. The same designs as those discussed previously were used. The BC_4_F_3_ ILs with the major QTL *qLW*20-1 were selected based on genotypes at the locus bin20-185 *via* PCR.

## Results

### Phenotypic Analysis of the Parents and Populations

**Figure 1:**
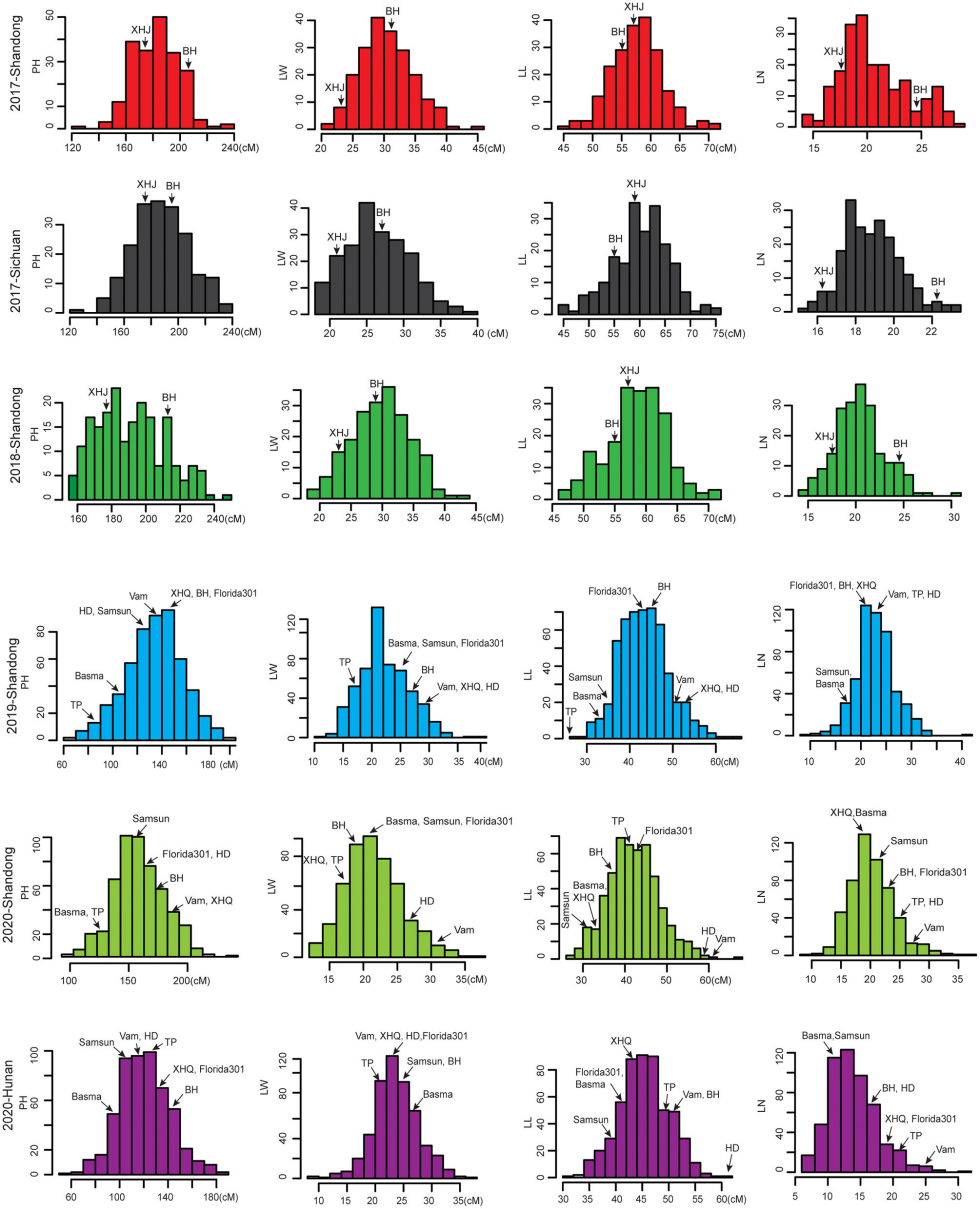
The frequency distributions of the four important agronomic traits in tobacco.

The phenotypic frequency distributions of four agronomic traits across different environments are shown in Figure 1. In the biparental RIL population, the two parental lines, Beinhart1000-1 (BH) and Xiaohuangjin1025 (XHJ), exhibited significantly different agronomic trait phenotypes in different environments. While the difference between the two parents varied according to the environment, XHJ had consistently greater LL, while BH had greater PH, LW, and LN in all the environments. Transgressive segregations of the four traits were observed in the RIL population, and their distributions were approximately normal. For the MAGIC population, the eight parental lines—Vam, XHQ, BH, Basma, Samsun, Florida301, TP, and HD—exhibited significantly different agronomic trait phenotypes in different environments. Basma and TP had lower PH values than the other six parents in the different environments. Moreover, compared with the other seven parents, Vam presented higher LNs in the different environments. The different parents showed different variations in LL and LW in the different environments. The MAGIC population showed large phenotypic variances across different environments for the four agronomic traits, with ranges of 55.83–235.00 cm for PH, 6.17–36.67 for LN, 27.08–67.50 cm for LL, and 8.17–39.50 cm for LW.

In addition, significant correlations were found between PH and the other traits in the RIL population (Supplementary Figure 1). However, the correlation between LL and LW was not significant, suggesting that the leaf shape (indicated by the LL/LW ratio) varied greatly within this population. As shown in Table 1, the variations in the four traits were all determined by genotype and environment. According to the plot, all traits had a moderate heritability of ~50%. These results indicated that the four agronomic traits were all quantitatively inherited in tobacco.

**Table 1:** Variance components and heritability for the four traits in the tobacco biparental population and MAGIC population in different environments.

**Population**	**Traits**	**Mean squares**				**Heritability (%)**	
		**Environment**	**Genotype**	**G × E**	**Error**	**Plot level**	**Genotypic mean level**
biparental population	PH	8019.46	1659.24	230.55	124.93	59.70	86.90
	LL	700.39	89.53	27.74	8.26	43.40	74.60
	LW	2069.62	77.69	15.79	9.32	48.40	81.20
	LN	398.44	23.26	6.38	1.91	46.60	77.00
MAGIC population	PH	376793.06	1835.34	506.13	195.94	44.12	76.41
	LL	23385.99	54.23	14.11	8.02	41.49	76.61
	LW	2804.38	163.23	30.05	15.12	52.64	83.13
	LN	965.65	71.89	16.41	6.48	49.15	79.94

### Construction of a High-Density Linkage map

Of the 56,693 polymorphic SNP markers, 17,788 SNP markers with <10% missing data were used to construct a linkage map. A total of 3,934 SNP markers were ultimately mapped onto the 24 high-density genetic LGs of tobacco (Supplementary Figure 2 and Supplementary Table 1). The total length of the LGs was 3920.43 cm, with a mean distance of 1.01 cm between adjacent markers. The LG length ranged from 100.09 cm to 255.58 cm. The most saturated LG was LG14, which had an intermarker distance of 0.81 cm, whereas LG18 had the largest distance of 1.34 cm between adjacent markers. The largest LG, LG01, harbored 252 markers, with a mean intermarker distance of 1.01 cm. The smallest LG, LG23, contained 91 markers, with a mean distance of 1.32 cm between adjacent markers (Supplementary Table 2).

### QTL Analysis

#### LM With a Biparental RIL Population

A total of 35 QTLs associated with the four agronomic traits were mapped onto 16 LGs in three different environments (Table 2). Among them, nine, nine, nine, and eight QTLs were found to be associated with PH, LL, LW, and LN, respectively. For PH, a total of nine QTLs (*qPH2-1, qPH4-1, qPH8-1, qPH11-1, qPH13-1, qPH13-2, qPH17-1, qPH18-1*, and *qPH19-1*) were identified under different conditions. They were distributed across LGs 2, 4, 8, 11, 13, 17, 18, and 19 and explained 3.49% to 47.04% of the total phenotypic variance. The BH alleles at all loci except *qPH4-1, qPH13-1*, and *qPH18-1* increased the trait values. One major QTL, *qPH17-1*, was repeatedly detected under different conditions and accounted for 7.06%−47.04% of the phenotypic variance. Nine QTLs (*qLL2-1, qLL4-1, qLL8-1, qLL12-1, qLL16-1, qLL17-1, qLL18-1, qLL20-1*, and *qLL22-1*) were detected for LL under different conditions, which were distributed across LGs 2, 4, 8, 12, 16, 17, 18, 20, and 22 and explained 5.39% to 19.81% of the total phenotypic variance. The BH alleles at all loci except *qLL2-1, qLL4-1, qLL18-1*, and *qLL20-1* increased the trait values. Another major QTL, *qLL17-1*, was repeatedly detected under different conditions and accounted for 5.67%−19.81% of the phenotypic variance. For LW, a total of nine QTLs (*qLW2-1, qLW4-1, qLW9-1, qLW11-1, qLW13-1, qLW17-1, qLW20-1, qLW21-2*, and *qLW23-1*) were identified under different conditions. They were distributed across LGs 2, 4, 9, 11, 13, 17, 20, 21, and 23 and explained 2.78% to 31.47% of the total phenotypic variance. The BH alleles at all loci, except *qLW11-1, qLW13-1, qLW17-1*, and *qLW20-1*, reduced the trait values. Another major QTL, *qLW20-1*, was repeatedly detected under different conditions and accounted for 19.24–31.47% of the total phenotypic variance. For LN, a total of eight QTLs (*qLN1-1, qLN4-1, qLN12-1, qLN12-2, qLN13-1, qLN17-1, qLN18-1*, and *qLN22-1*) were identified under different conditions. They were distributed across LGs 1, 4, 12, 13, 17, 18, and 22 and explained 4.79% to 20.16% of the total phenotypic variance. The BH alleles at all loci, except *qLN4-1* and *qLN22-1*, increased the trait values.

**Table 2:** Summary of QTLs affecting the four important agronomic traits in tobacco across different environments using the LM method.

**Trait**	**QTL**	**LG^a^**	**Sichuan in 2017**				**Shandong in 2017**				**Shandong in 2018**				**BLUE** ^f^			
			**Pos.^b^**	**LOD^c^**	**Add^d^**	**PVE (%)^e^**	**Pos**.	**LOD**	**A**	**PVE (%)**	**Pos**.	**LOD**	**A**	**PVE (%)**	**Pos**.	**LOD**	**A**	**PVE (%)**
PH	*qPH2-1*	2	/^g^	/	/	/	/	/	/	/	/	/	/	/	142	4.83	−1.14	3.49
	*qPH4-1*	4	105	22.17	−9.74	18.09	105	5.39	−4.50	7.06	101	12.25	−6.76	9.17	101	13.66	−5.72	10.96
	*qPH8-1*	8	59	7.06	0.38	4.88	/	/	/	/	/	/	/	/	/	/	/	/
	*qPH11-1*	11	/	/	/	/	78	4.55	3.79	5.99	/	/	/	/	77	6.84	3.11	5.03
	*qPH13-1*	13	152	16.99	−8.22	12.81	/	/	/	/	/	/	/	/	/	/	/	/
	*qPH13-2*	13	167	7.06	5.05	4.74	/	/	/	/	/	/	/	/	/	/	/	/
	*qPH17-1*	17	51	27.19	11.06	23.13	51	19.72	9.21	30.04	51	42.77	15.42	47.04	51	41.09	11.82	45.92
	*qPH18-1*	18	/	/	/	/	/	/	/	/	157	5.27	−4.27	3.63	/	/	/	/
	*qPH19-1*	19	/	/	/	/	/	/	/	/	87	5.75	4.34	3.94	/	/	/	/
LL	*qLL2-1*	2	129	5.65	−0.44	6.43	/	/	/	/	/	/	/	/	63	4.62	−0.95	5.39
	*qLL4-1*	4	/	/	/	/	/	/	/	/	/	/	/	/	81	5.71	−1.06	6.05
	*qLL8-1*	8	67	8.20	1.81	9.93	/	/	/	/	/	/	/	/	/	/	/	/
	*qLL12-1*	12	/	/	/	/	170	5.79	1.30	7.04	/	/	/	/	/	/	/	/
	*qLL16-1*	16	/	/	/	/	121	9.98	1.77	13.26	/	/	/	/	/	/	/	/
	*qLL17-1*	17	41	9.89	2.00	11.70	41	4.74	1.19	5.67	41	13.73	2.35	19.81	41	13.73	1.76	15.82
	*qLL18-1*	18	/	/	/	/	/	/	/	/	152	7.20	−1.60	9.48	152	8.88	−1.36	9.58
	*qLL20-1*	20	170	8.68	−1.98	10.65	/	/	/	/	/	/	/	/	180	8.58	−1.42	9.45
	*qLL22-1*	22	/	/	/	/	0	4.94	1.21	5.94	/	/	/	/	/	/	/	/
LW	*qLW2-1*	2	/	/	/	/	/	/	/	/	53	6.48	−1.32	8.28	/	/	/	/
	*qLW4-1*	4	76	5.93	−1.15	6.22	/	/	/	/	/	/	/	/	73	5.08	−0.68	2.97
	*qLW9-1*	9	/	/	/	/	/	/	/	/	/	/	/	/	133	11.22	−1.08	6.65
	*qLW11-1*	11	54	12.36	1.75	14.11	/	/	/	/	/	/	/	/	/	/	/	/
	*qLW13-1*	13	/	/	/	/	/	/	/	/	/	/	/	/	188	4.91	0.68	2.78
	*qLW17-1*	17	/	/	/	/	/	/	/	/	53	13.86	2.05	20.09	54	27.94	1.79	19.72
	*qLW20-1*	20	185	20.73	2.45	26.29	188	21.18	2.43	28.09	185	14.10	2.01	19.24	185	38.87	2.37	31.47
	*qLW21-1*	21	/	/	/	/	/	/	/	/	/	/	/	/	29	7.80	−0.86	4.47
	*qLW23-1*	23	/	/	/	/	16	4.64	−0.36	5.81	/	/	/	/	/	/	/	/
LN	*qLN1-1*	1	/	/	/	/	/	/	/	/	41	4.73	0.21	4.79	/	/	/	/
	*qLN4-1*	4	/	/	/	/	/	/	/	/	/	/	/	/	101	5.79	−0.44	6.20
	*qLN12-1*	12	1	5.83	0.49	12.79	/	/	/	/	/	/	/	/	/	/	/	/
	*qLN12-2*	12	/	/	/	/	157	9.31	1.19	11.83	157	13.22	1.06	15.08	157	16.15	0.82	20.16
	*qLN13-1*	13	/	/	/	/	/	/	/	/	66	10.06	0.91	10.70	60	8.28	0.53	9.11
	*qLN17-1*	17	/	/	/	/	46	11.69	1.43	15.39	46	5.21	0.64	5.34	51	4.83	0.41	5.14
	*qLN18-1*	18	/	/	/	/	59	5.48	0.92	6.85	59	6.44	0.70	6.68	/	/	/	/
	*qLN22-1*	22	/	/	/	/	/	/	/	/	100	11.49	−0.87	12.09	99	4.52	−0.35	5.03

a *Linkage group*.

b *Linkage group (cM) of the LOD peak*.

c *LOD, the logarithm of odds*.

d *Additive effects*.

e *PVE, phenotypic variation explained*.

f *BLUE, best linear unbiased estimation*.

g */ = Absent*.

**Figure 2:**
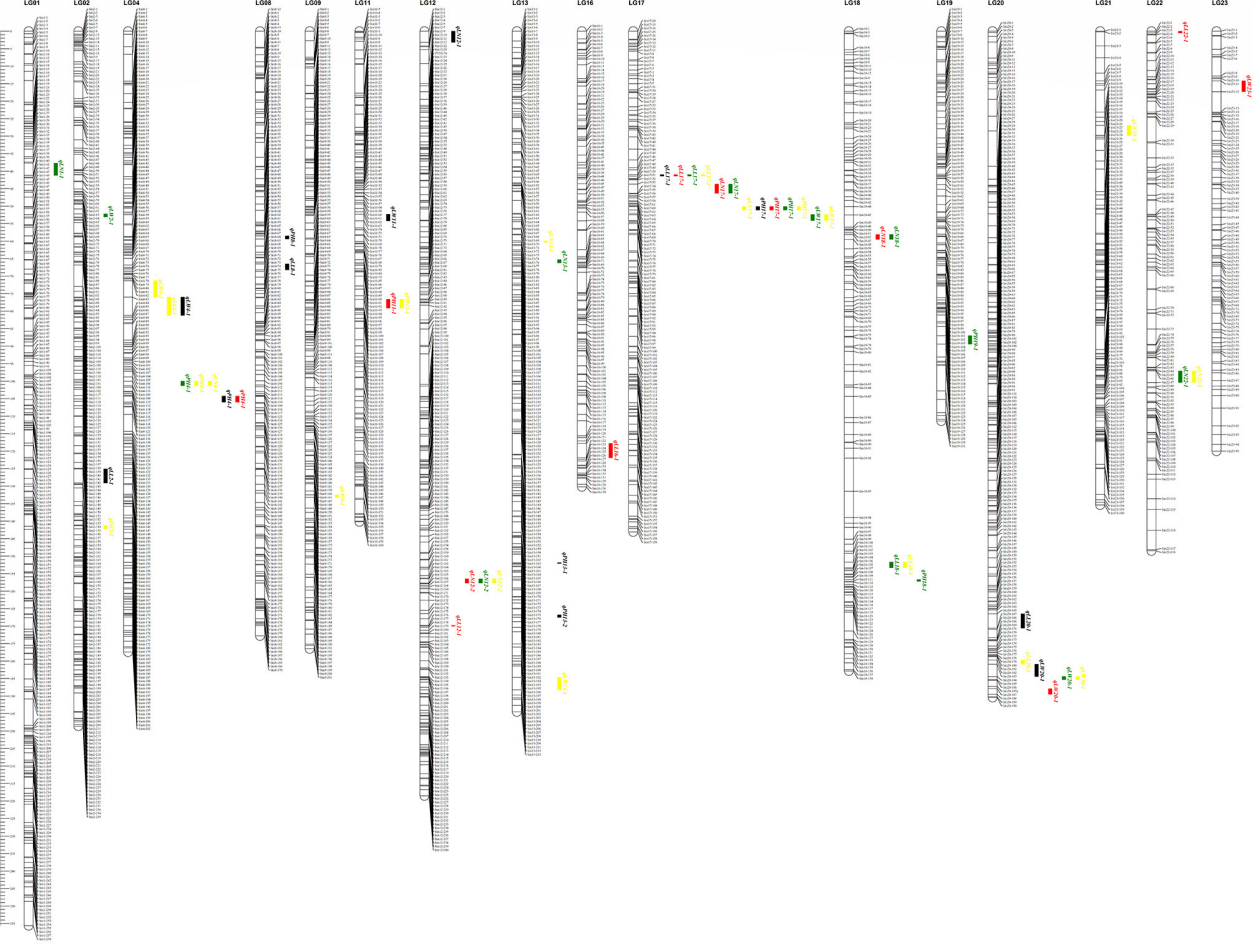
The positions of QTL affect the four important agronomic traits in tobacco by the linkage mapping method. The black box represents QTL detected at Sichuan in 2017; the red box represents QTL detected at Shandong in 2017; the green box represents QTL detected at Shandong in 2018; and the yellow box represents QTL detected for BLUE.

As shown in Figure 2, many regions were found to harbor two or more closely linked QTLs for different traits. For instance, *qPH17-1, qLL17-1, qLW17-1*, and *qLN17-1* were mapped to the adjacent region based on LG17, and these QTLs were tightly linked to markers bin17-48 and bin17-70. These results were in agreement with those of a correlation analysis of phenotypic traits in the RIL population. Two major QTLs (*qPH17-1* and *qLW20-1*) were repeatedly identified using the LM method in the three environments. For PH, one major QTL, named *qPH17-1*, was mapped to the same chromosomal region from bin17-66 to bin17-67. This QTL explained 23.13% to 47.04% of the phenotypic variation with positive additive effects under different conditions. For LW, the other major QTL, named *qLW20-1*, was mapped to a similar chromosomal region from bin20-183 to bin20-188. This QTL explained 19.24% to 31.47% of the phenotypic variation with positive additive effects under different conditions.

#### AM via a MAGIC Population

**Figure 3:**
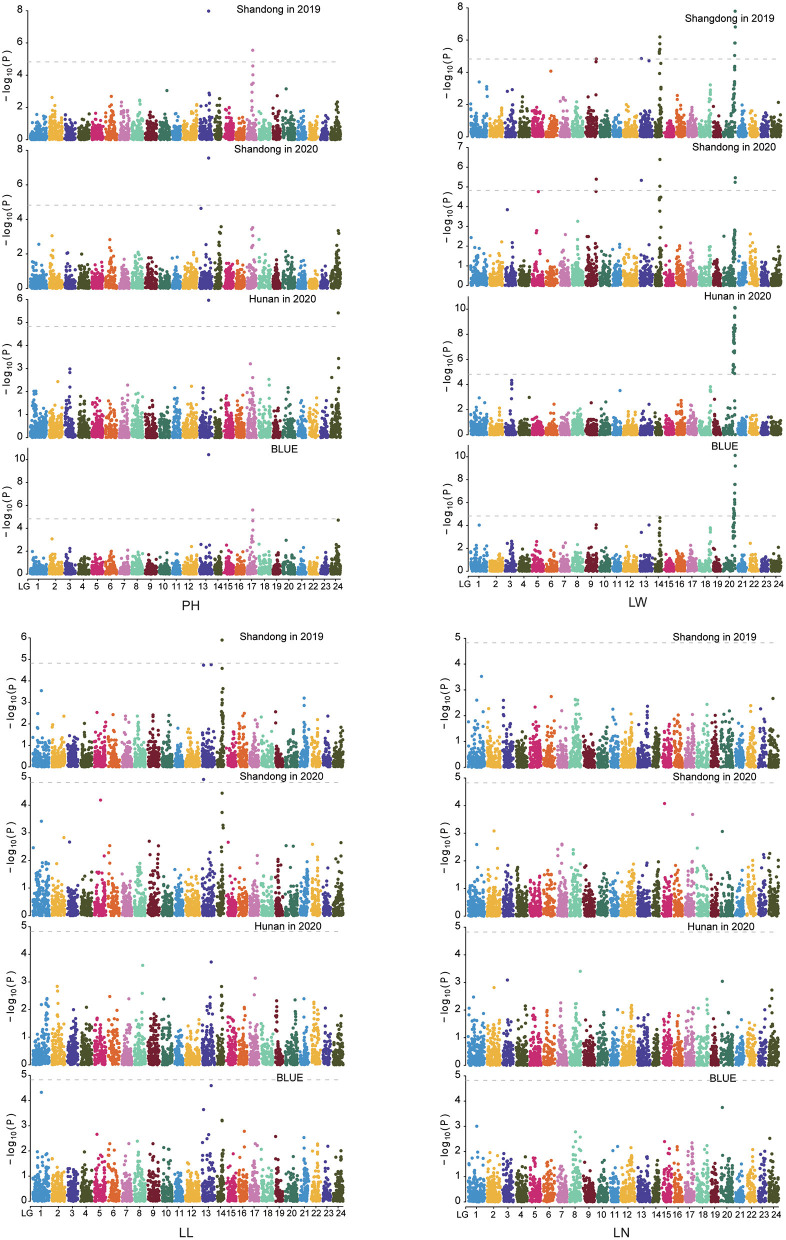
Association mapping for the four important agronomic traits in tobacco using the MAGIC population. The x- and y-axes represent the genetic position of the 12 linkage groups, respectively, as well as the negative log10 *P-value*. The horizontal solid line indicates the genome-wide significance threshold *P* = 1.52 × 10^−5^.

To investigate the genetic relationship among MAGIC lines, we performed a genetic analysis based on 3,282 SNP markers (Supplementary Table 3). The results suggested that the eight-way populations showed no clear population structure (Supplementary Figure 3). Then, using the MAGIC population, we carried out GWAS of the four agronomic traits, namely, PH, LW, LL, and LN. A total of nine QTLs for PH, LW, and LL were identified by the MLM method (Figure 3, Table 3, and Supplementary Figure 4). Among these QTLs, three association signals were identified for PH, especially *qPH13-3*, which was repeatedly detected for PH in all environments. Another two QTLs, *qPH17-2* and *qPH24-1*, were identified only in Shandong in 2019 and in Hunan in 2020, respectively. For LW, four QTLs, namely, *qLW20-1, qLW14-1, qLW13-2*, and *qLW9-2*, were repeatedly identified in at least two environments (Shandong in 2019 and 2020), and *qLW20-1* was identified in all the environments. Additionally, two QTLs (*qLL13-1* and *qLL14-1*) for LL were mapped onto LGs 13 and 14, respectively.

**Table 3:** Summary of QTLs affecting the four important agronomic traits in tobacco in different environments using the AM method.

**Trait**	**QTL**	**SNP**	**Shandong in 2019**			**Shandong in 2020**			**Hunan in 2020**			**BLUE**		
			**Pos^a^**	***p***	**Effect**	**Pos**	***p***	**Effect**	**Pos**	***p***	**Effect**	**Pos**	***p***	**Effect**
PH	*qPH13*-3	bin13-161	140	1.06E-08	13.40	140	2.79E-08	12.40	140	1.08E-06	10.30	140	3.70E-11	11.70
	*qPH17-2*	bin17-101	90	2.84E-06	−6.40	/	/	/	/	/	/	90	2.54E-06	−5.00
	*qPH24-1*	bin24-103	/	/	/	/	/	/	100	3.83E-06	6.00	/	/	/
LW	*qLW20-1*	bin20-185	185	1.62E-08	−2.90	185	5.81E-06	−2.20	185	7.44E-11	−4.10	185	7.69E-11	−2.70
	*qLW14-1*	bin14-108	75	6.39E-07	1.70	75	9.20E-06	1.40	/	/	/	/	/	/
	*qLW13-2*	bin13-31	22	1.40E-05	1.50	22	4.62E-06	1.50	/	/	/	/	/	/
	*qLW9-2*	bin9-186	163	1.49E-05	1.20	163	4.03E-06	1.20	/	/	/	/	/	/
LL	*qLL14-1*	bin14-108	75	1.26E-06	2.20	/	/	/				/	/	/
	*qLL13-1*	bin13-31	/	/	/	22	1.17E-05	1.10				/	/	/

a *Genetic position (cM) of the LOD peak*.

### Candidate Gene Prediction of Major QTLs

The candidate genes for the three major QTLs (*qPH13-3, qPH17-1*, and *qLW20-1*) were predicted based on the physical positions of the tightly linked markers. The *qPH13-3* was tightly linked to the marker bin13-161 by the AM method; this QTL was determined to be located on Nt17:170,496,603 bp based on the K326 reference genome reported by Edwards et al. (2017). Comparative mapping of the K326 reference genome predicted the existence of a total of 26 genes from 169.99Mb to 170.99Mb on Nt17. The polymorphisms of the 26 candidate genes were analyzed between the different parents using the resequencing method. As shown in Supplementary Table S4, non-synonymous mutations existed in six genes among the 26 candidate genes. In sum, one gene (*Nitab4.5_0002347g0190.1*) encoding a protein with high homology to gibberellin 2-beta-dioxygenase 8 (*GA2OX8*) in *Arabidopsis thaliana* (Li Y. et al., 2019) was considered as the candidate gene for *qPH13-3*. Another QTL involved in PH, *qPH17-1*, was mapped by the AM method to LG 17 between bin17-66 and bin17-67. Further analysis indicated that the markers bin17-66 and bin17-67 were located at Nt17: 147,923,432 bp and Nt17: 150,216,340 bp. There were 68 candidate genes in this target region. For LW, the major QTL *qLW20-1* was tightly linked to the marker bin20-185 (Nt23: 997,702 bp). There were 13 candidate genes from 0.50Mb to 1.50Mb on chromosome 23. The expression level and sequence polymorphisms of the 13 candidate genes were analyzed between the different parents. There was no difference between parental lines, XHJ and BH, for the expression of the 13 candidate genes, and non-synonymous mutations existed in four of the 13 candidate genes. Among these genes, one gene (*Nitab4.5_0000798g0140.1*) encoding a protein with high homology to auxin response factor 9 (*ARF9*) in *Arabidopsis thaliana* (Remington et al., 2004) was considered the candidate gene for *qLW20-1* (Supplementary Table 4).

### Validation and Utilization of the Major QTL *qLW20-1* for Improvement of LW in Tobacco

**Figure 4:**
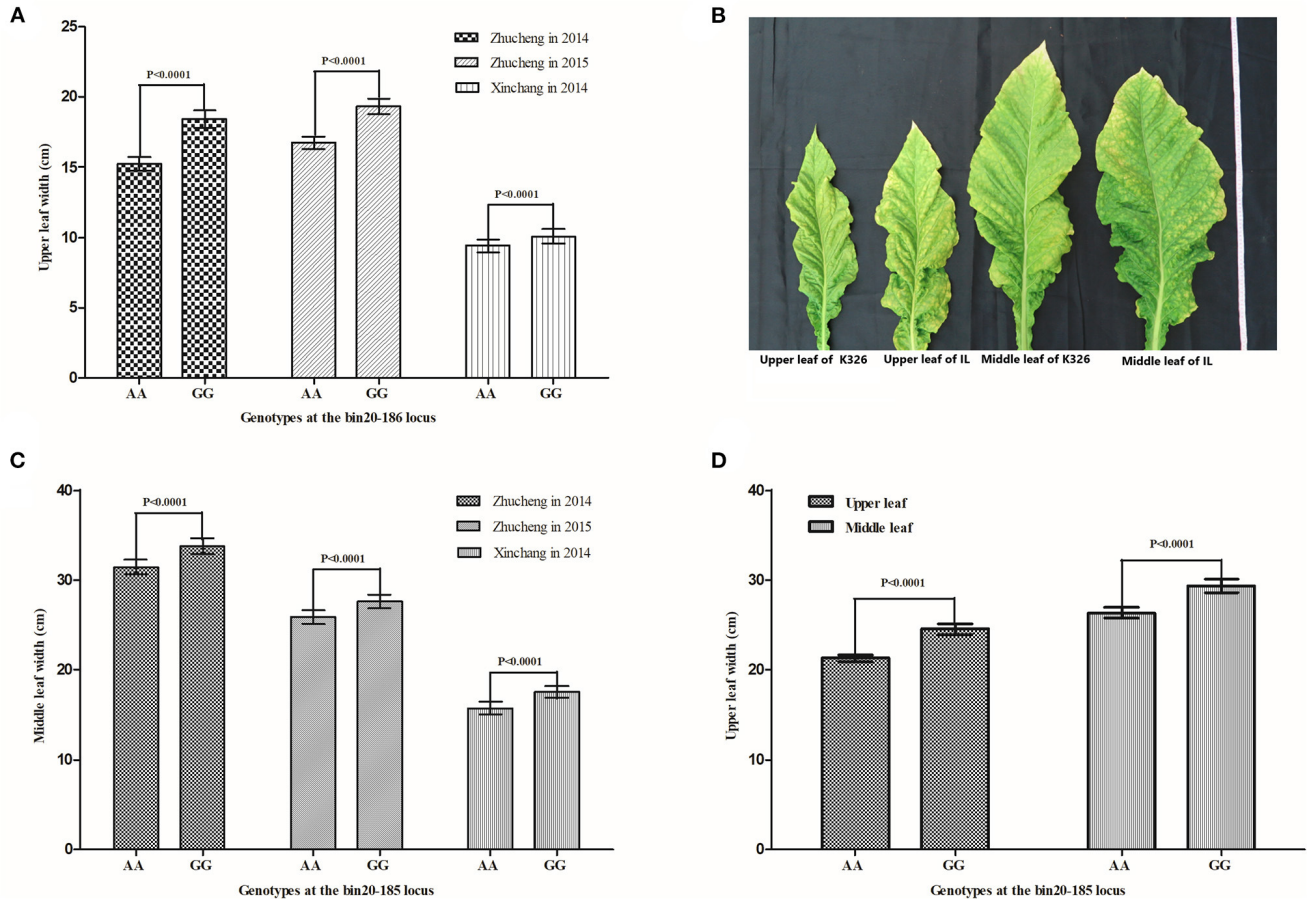
Improvement of LW in tobacco by MAS based on the major QTL *qLW20-1*. **(A)** A comparison of upper LW between different genotypes in the tobacco collection in different environments. **(B)** A comparison of middle LW between different genotypes in the tobacco collection in different environments. **(C)** The performance of upper and middle leaves between the donor parent K326 and the line anchored to the major QTL controlling tobacco, LW. **(D)** A comparison of upper and middle LWs between the donor parent K326 and the line that anchored the major QTL controlling tobacco LW. Statistical analysis of LW between different lines based on different genotypes (AA and GG) at the locus bin20-185. The error bars indicate SD. Significant tests are carried out using student's *t*-tests.

A major QTL, *qLW20-1*, associated with the LW trait in tobacco, was repeatedly detected in all environments and was tightly linked to the SNP marker bin20-185 (Tables 2, 3). The 211 tobacco accessions were genotyped at the *qLW20-1* locus and were divided into two groups (AA and GG) based on the genotypes of the flanking marker bin20-185 (Supplementary Table 5). The accessions in the AA group presented the same genotypes with XHJ at the *qLW20-1*, while the accessions in the GG group presented the same genotypes with BH at the *qLW20-1*. The values for LW in three different environments were obtained for all the genotyped accessions. As shown in Figures 4A,B, there was a significant difference (*P* < 0.0001) in the LW trait between the GG group (comprising 114 tobacco accessions) and the AA group (comprising 97 tobacco accessions). The average values of LW were significantly higher in the GG group than in the AA group in different environments. These results indicated a strong correlation between the genotype of the marker bin20-185 and the LW trait. A new BC_4_F_3_ population derived from the flue-cured tobacco K326 (recurrent parent and AA genotype) and the oriental tobacco Samsun (donor parent and GG genotype) was constructed. As shown in Figures 4C,D, tobacco accessions with GG genotypes at the bin20-185 site exhibited significantly greater LW than the recurrent parent K326.

## Discussion

### Mapping for Complex Traits in Tobacco

In comparison to other important crop species, only a few studies of QTL mapping for complex traits in tobacco have been reported due to the following reasons. First, as a member of the Solanaceae family, tobacco is an allopolyploid species (2n = 48) with a large genome (4.5 Gb) (Sierro et al., 2014). During its evolution, tobacco experienced a genetic bottleneck, resulting in the very low diversity of tobacco accessions present today. Second, suitable genetically different populations for complex trait mapping in tobacco are lacking. To date, only a few studies involving biparental permanent populations, such as RIL and DH populations, have been reported for QTL mapping. Third, many QTL studies are dependent on traditional molecular markers, such as SSRs, amplified fragment length polymorphisms (AFLPs), and restriction fragment length polymorphisms (RFLPs), which have a lower density than SNPs, resulting in insufficient resolution during genetic mapping (Julio et al., 2006; Tan et al., 2012; Cheng et al., 2015; Sun et al., 2018; Zhang et al., 2018). In this study, we constructed a high-density SNP linkage map of tobacco based on a BH/XHJ RIL for genome-wide QTL mapping. Then, major QTLs related to four important agronomic traits were accurately identified by the use of a biparental RIL and a MAGIC population. Finally, the LW of the main tobacco variety K326 was successfully improved *via* MAS. Our results indicate that combining a high-density SNP linkage map with a different genetic population can be a highly successful strategy for the improvement of complex traits in tobacco.

### Comparison of QTL Mapping Results Between the MAGIC Population and the Biparental Population in Tobacco

MAGIC populations are considered to be more effective and powerful for QTL mapping, gene discovery, and breeding of many crop species (Cavanagh et al., 2008; Bandillo et al., 2013; Meng et al., 2016; Campanelli et al., 2019). To date, QTL mapping involving MAGIC populations has not been reported in tobacco. In this study, QTL mapping of four agronomic traits in tobacco was performed by the use of a biparental population and a MAGIC population. Compared to the biparental population, the MAGIC population showed wider phenotypic variances (Figure 1). This suggested that the MAGIC population provides breeders with a greater opportunity to select elite lines for breeding. However, the number of QTLs identified in the biparental population was greater than that in the MAGIC population. There are several plausible reasons for this. First, in our study, the *P*-value (P_adjusted_ = 0.05/number of markers) of the GWAS was adjusted by the Bonferroni correction method, which is rather strict for multiple comparisons and makes it easier to ignore meaningful sites. To reduce the false-negative discovery rate, we adopted a more relaxed threshold, especially for complex traits (Li C. et al., 2019). Second, the use of more parents for developing a population usually implies that the frequencies of different alleles are lower and uneven across alleles, which reduces the power for detecting QTLs with weak effects (Meng et al., 2016). Third, the SNP markers for AM were derived from these polymorphic markers and mapped to LGs using a biparental population instead of the mapping of all eligible markers in the MAGIC population. The MAGIC population has lower detection power than the biparental population for minor-effect QTLs given similar numbers of markers. However, the MAGIC population is very effective for major QTL detection in tobacco. One major QTL, *qLW20-1*, identified *via* the biparental population, was also repeatedly detected *via* the MAGIC population, and one major QTL, *qPH13-3*, was identified specifically in the different environments *via* the MAGIC population. Therefore, it is necessary for the MAGIC population to improve its mapping power and resolution by expanding marker coverage in the future.

### Major QTLs for PH and LW in Tobacco

According to the results of LM and AM, a total of 43 QTLs were detected for four important agronomic traits in the different populations in different environments. Among these QTLs, two or more QTLs for different traits were closely linked and located in similar chromosomal regions. These results implied that some agronomic traits were closely related to each other in tobacco. Three major QTLs, *qPH13-3, qPH17-1*, and *qLW20-1*, were repeatedly detected under different conditions. Using 614 SSR markers, Tong et al. (2012) reported four QTLs associated with PH, internode length, and the width of the largest waste leaf, explaining 15%−20% of the phenotypic variance. According to AM, *qPH13-3* in this study colocalized with *qPH17-1* and *qLWL17* reported by Tong et al. (2012) and was tightly linked to the marker bin13-161. This marker (Nt17:170,496,603 bp) was located in the first intron region of the gene *Nitab4.5_0002347g0190.1*. This gene encodes a protein with high homology to gibberellin 2-beta-dioxygenase 8 (*GA2OX8*) in *Arabidopsis thaliana*, and this protein acts specifically on C-20 gibberellins (Li C. et al., 2019). Therefore, *Nitab4.5_0002347g0190.1* is considered a candidate gene for *qPH13-3* and may play an important role in determining tobacco height. For LW, *qLW20-1* may be a novel major QTL controlling leaf development in tobacco because it was identified in similar genomic regions in the different environments through combinations of LM and AM methods. In this study, *qLW20-1* was indicated to be tightly linked to marker bin20-185. In the target region, one gene (*Nitab4.5_0000798g0140.1*) encoded a protein with high homology to auxin response factor 9 (*ARF9*) in *Arabidopsis thaliana* (Remington et al., 2004) and was considered the candidate gene for *qLW20-1*. In the *Arabidopsis thaliana*, the orthologous gene (*AT4G23980*) encodes a transcription factor that controls the expression of the large set of auxin-dependent genes that mediate hormone-dependent growth and development. Therefore, it is possible that *Nitab4.5_0000798g0140.1* controls the expression of auxin-related genes, which govern LW in tobacco. Identification of candidate genes and functional research of these stably expressed QTLs are being carried out.

### Potential of *qLW20-1* for Breeding in Tobacco

The discovery of new QTLs can provide more choices for breeding new varieties using MAS (Cheng et al., 2013; Wang et al., 2014; Yin et al., 2015; Zhang et al., 2017). In the current study, we found that 114 varieties with the same allele (GG) as BH at the major QTL *qLW20-1* showed significant improvements in the LW trait. The results indicated that the GG genotype at the major gene locus played an important role in controlling the development of LW, and the responsible alleles have been widely incorporated into modern cultivated tobacco varieties. To improve the LW of K326 (a major flue-cured tobacco variety grown in China and the USA), we developed a set of introgression lines in the K326 genetic background from a cross between K326 and Samsun. Based on the information from the flanking SNP marker bin20-185, we selected 71 introgression lines (GG genotype). The average values of the upper LW and middle LW of the GG genotype introgression lines were significantly higher than those of the AA genotype. Therefore, the identification of the tightly linked molecular marker bin20-185 provides an opportunity for utilizing this genetic region for tobacco breeding more extensively and effectively.

## Conclusion

Overall, in this study, using the biparent and MAGIC populations, we mapped QTLs for PH, LW, LL, and LN in tobacco. A total of 43 QTLs, including three major QTLs related to different agronomic traits, were detected by the combination of LM and AM. These QTLs and markers tightly linked to the traits of interest in this study may be useful for the improvement of agronomic traits in tobacco *via* MAS.

## Data Availability Statement

The data presented in the study are deposited in the Genome Variation Map in National Genomics Data Center, China National Center for Bioinformation/Beijing Institute of Genomics, Chinese Academy of Sciences, accession number GVM000327 RIL and GVM000326 MAGIC.

## Author Contributions

LC and AY designed the experiment. YL, GY, YS, ZJ, DL, CJ, XP, JY, ZL, JZ, YP, LW, ZX, and QF performed the experiment. HS, LC, and MR analyzed experimental data. LC, HS, and YL wrote the manuscript. All authors read and approved the manuscript.

## Funding

This work was supported by grants from the Agricultural Science and Technology Innovation Program (ASTIP-TRIC01).

## Conflict of Interest

The authors declare that the research was conducted in the absence of any commercial or financial relationships that could be construed as a potential conflict of interest.

## Publisher's Note

All claims expressed in this article are solely those of the authors and do not necessarily represent those of their affiliated organizations, or those of the publisher, the editors and the reviewers. Any product that may be evaluated in this article, or claim that may be made by its manufacturer, is not guaranteed or endorsed by the publisher.

## Supplementary Material

Supplementary Figure 1 Pairwise correlation values among four agronomic traits in the RIL population of tobacco.

Supplementary Figure 2 The high-density SNP linkage map of tobacco based on a BH/XHJ recombinant inbred line population.

Supplementary Figure 3 Phylogenetic trees of the MAGIC lines.

Supplementary Figure 4 Q-Q plots for four agronomic traits across different environments.

Supplementary Table 1 The information from SNP markers was mapped on 24 linkage groups of tobacco.

Supplementary Table 2 The statistics of the high-density single nucleotide polymorphism-based genetic maps in the BH/XHJ population from the 430K SNP genotyping assay.

Supplementary Table 3 The information of SNP markers for association mapping using MAGIC population.

Supplementary Table 4 The list of candidate genes for the three major QTLs is *qPH13-3, qPH17-1*, and *qLW20-1*.

Supplementary Table 5 The list of tobacco accessions used for validation and utilization of the major QTL *qLW20-1*.
