# Supplementary figures and images for: Identification of QTLs Associated With Agronomic Traits in Tobacco via a Biparental Population and an Eight-Way MAGIC Population
Source: Front Plant Sci. 2022 Jun 6;13:878267. doi: 10.3389/fpls.2022.878267 (PMC9207565; doi:10.3389/fpls.2022.878267)

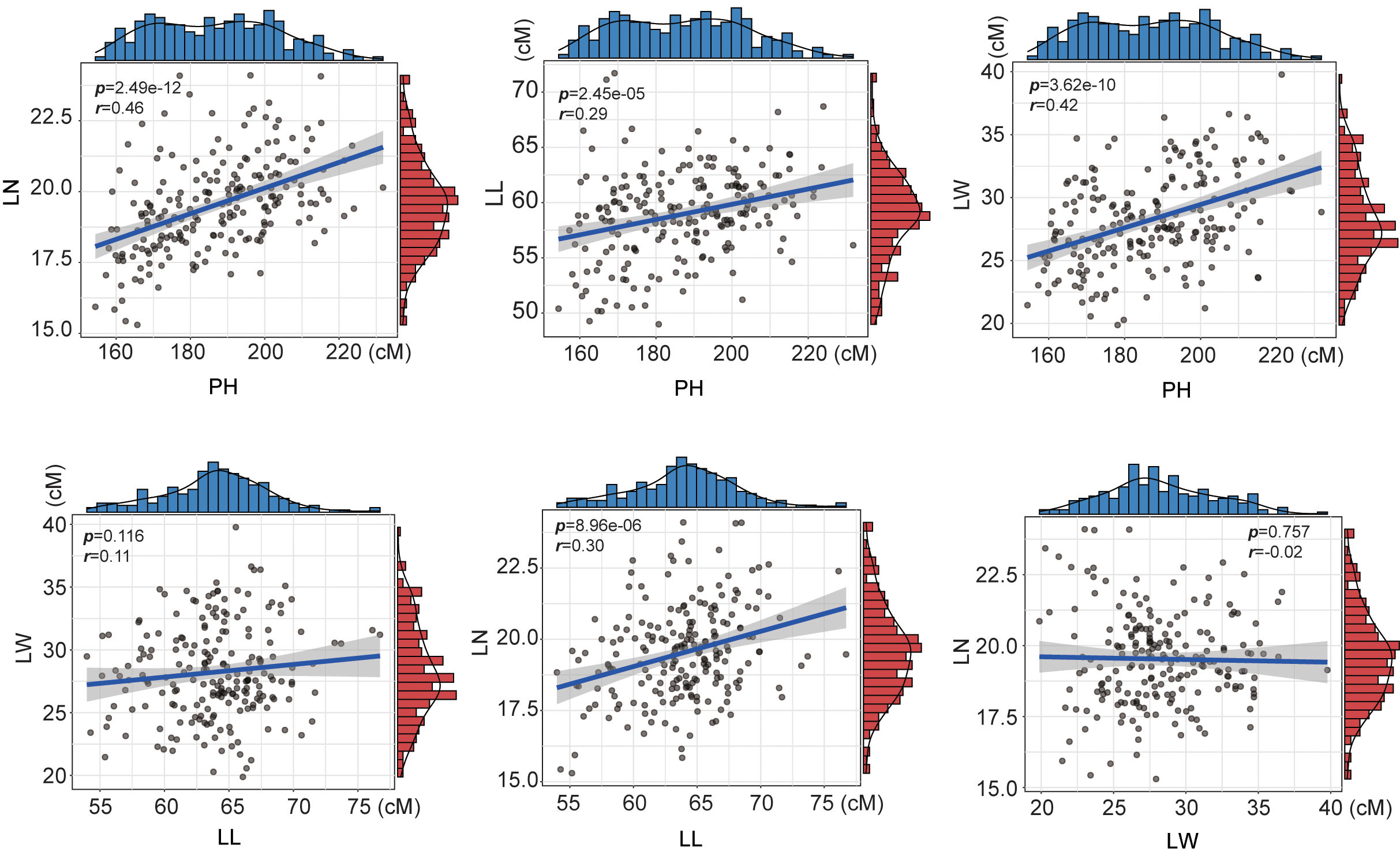

Supplement: Supplementary Figure 1 — Pairwise correlation values among four agronomic traits in the RIL population of tobacco. [file Image_1.TIF]

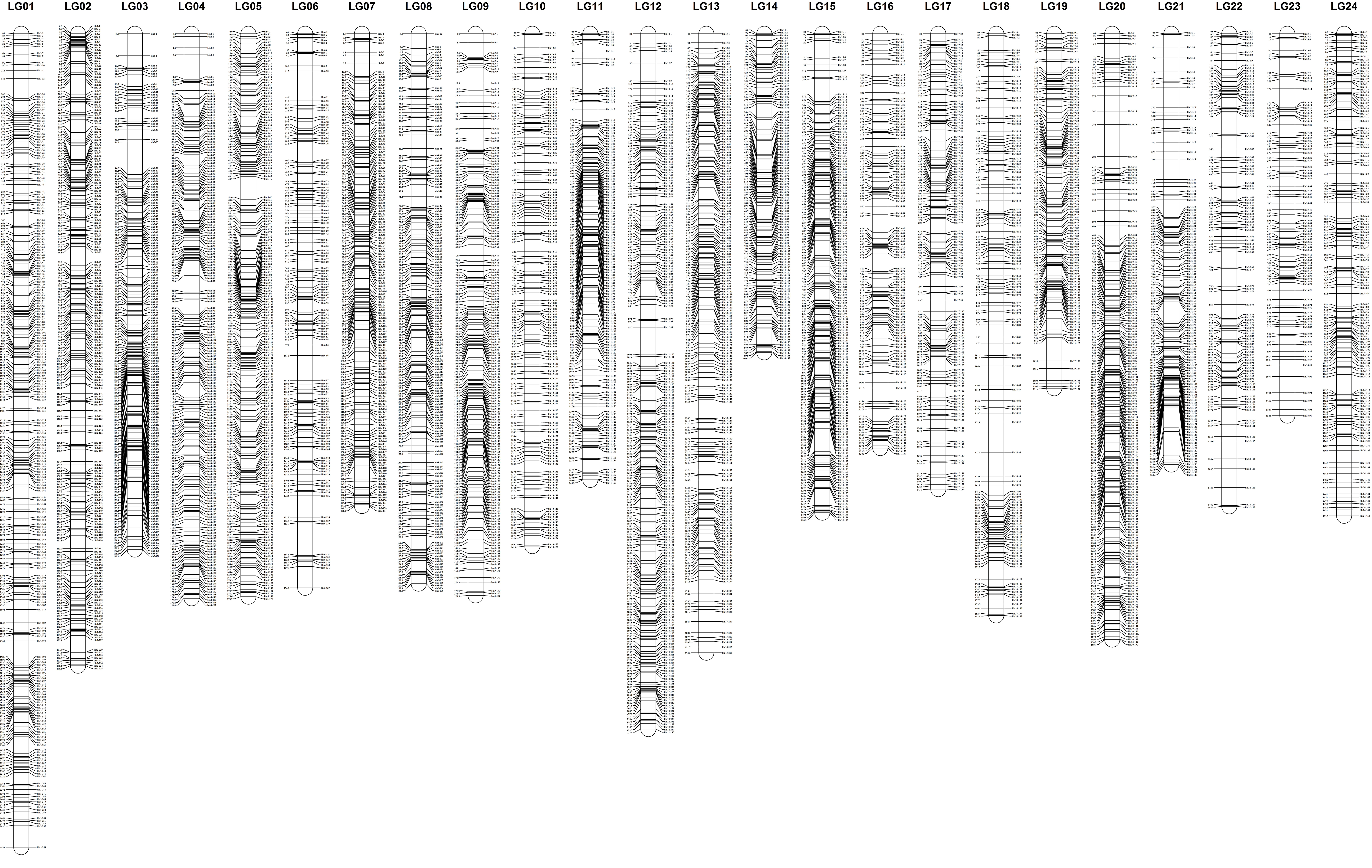

Supplement: Supplementary Figure 2 — The high-density SNP linkage map of tobacco based on a BH/XHJ recombinant inbred line population. [file Image_2.TIF]

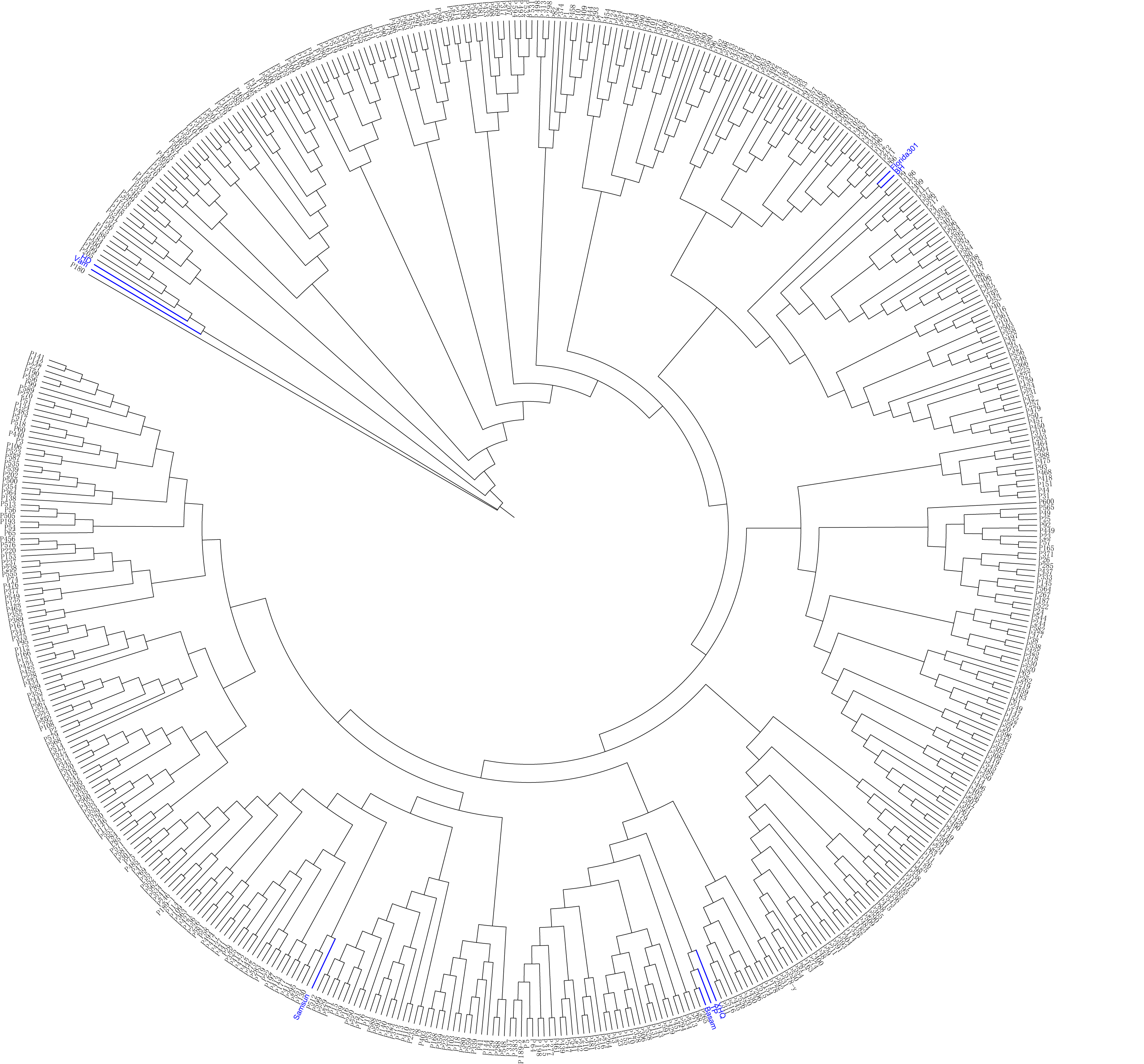

Supplement: Supplementary Figure 3 — Phylogenetic trees of the MAGIC lines. [file Image_3.TIF]

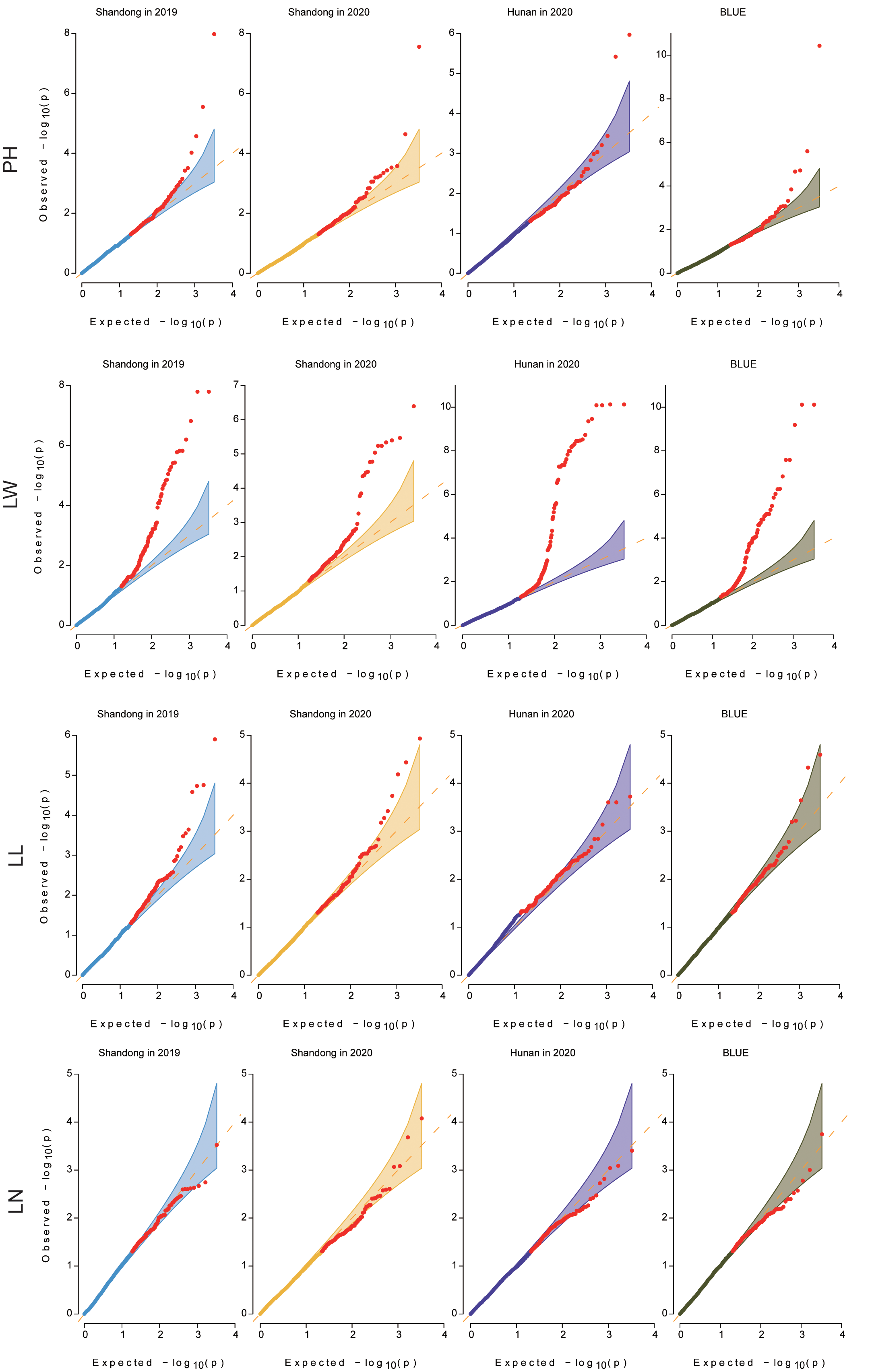

Supplement: Supplementary Figure 4 — Q-Q plots for four agronomic traits across different environments. [file Image_4.TIF]

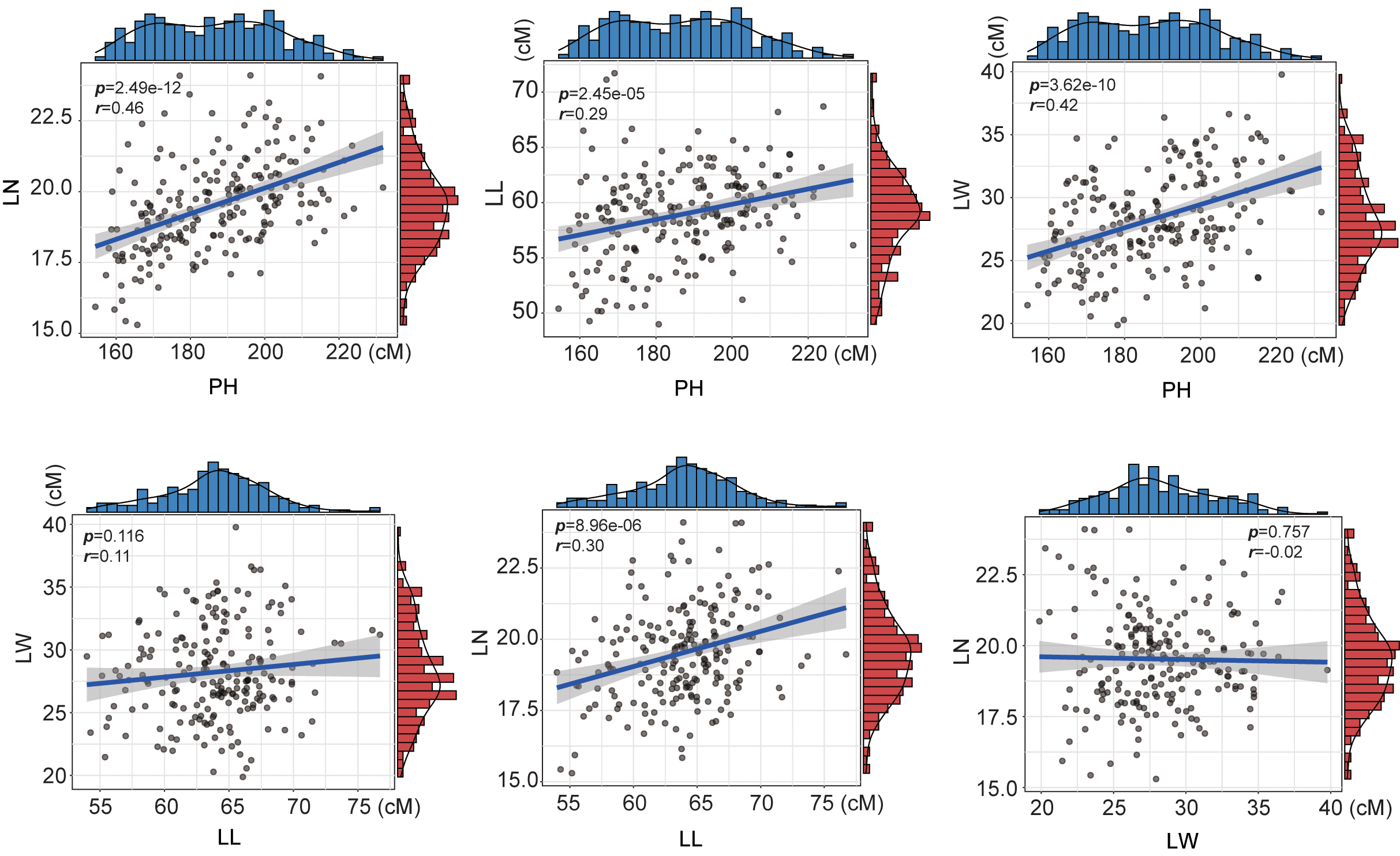

Supplement: Supplementary file 5 [file Image_5.TIF]

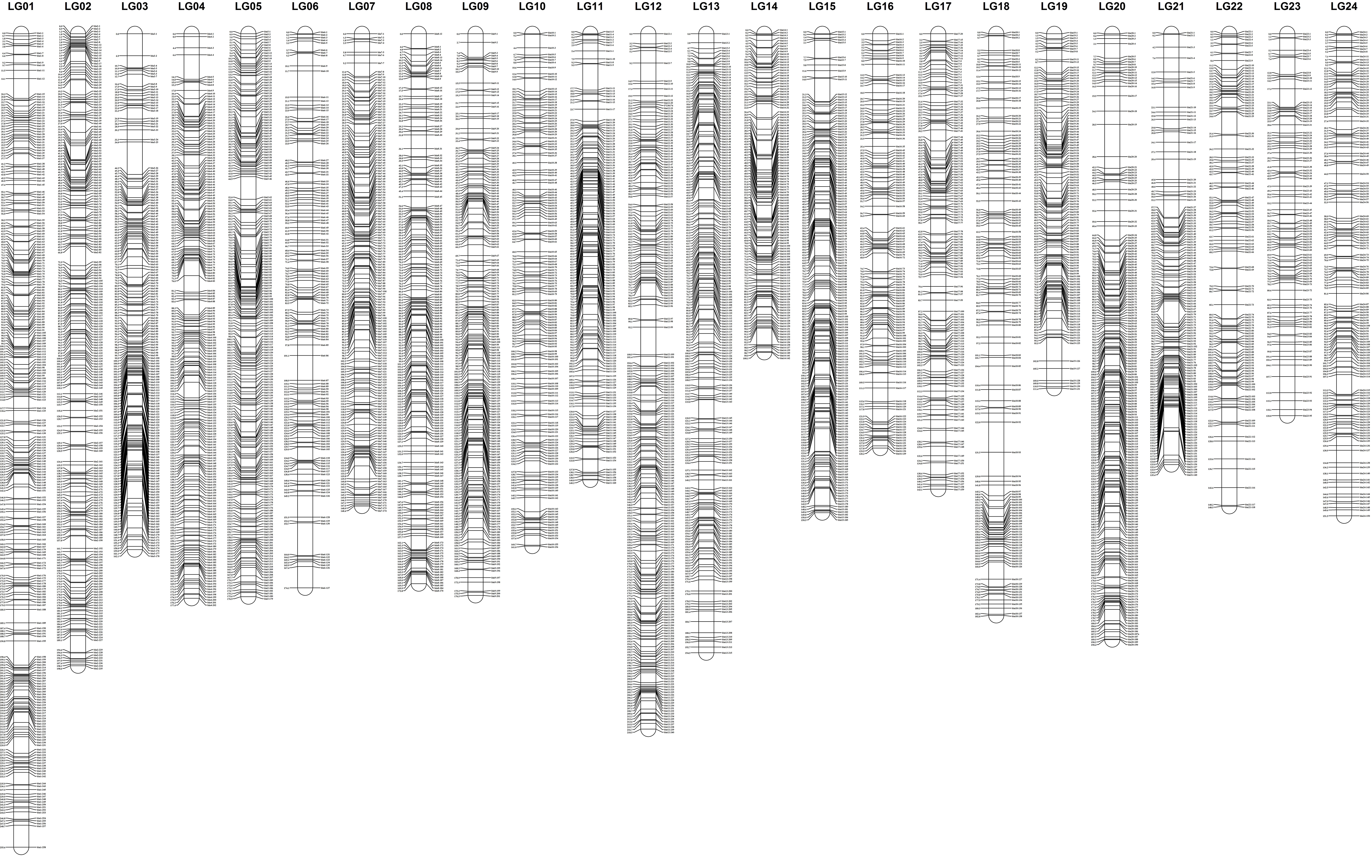

Supplement: Supplementary file 6 [file Image_6.TIF]

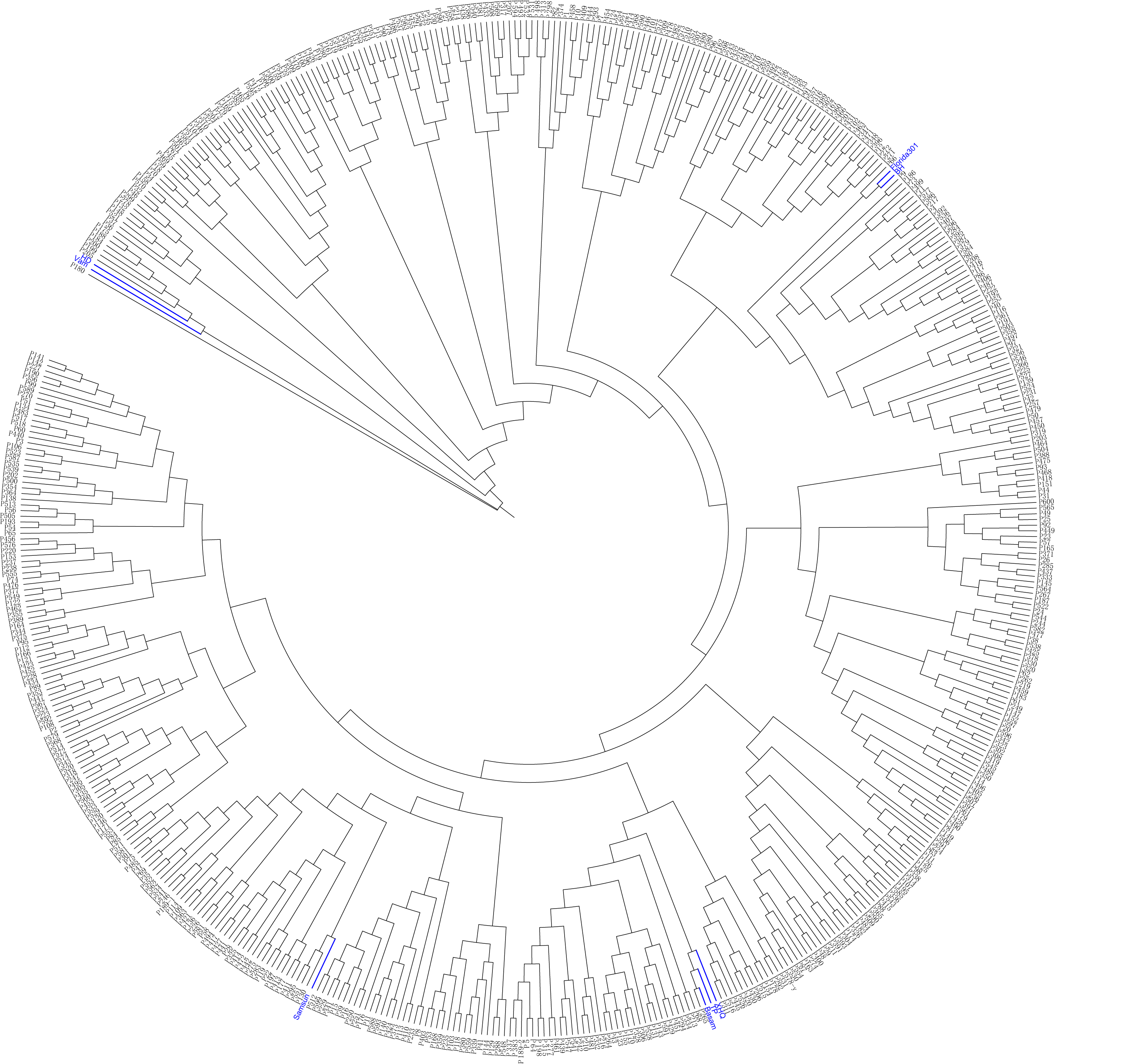

Supplement: Supplementary file 7 [file Image_7.TIF]

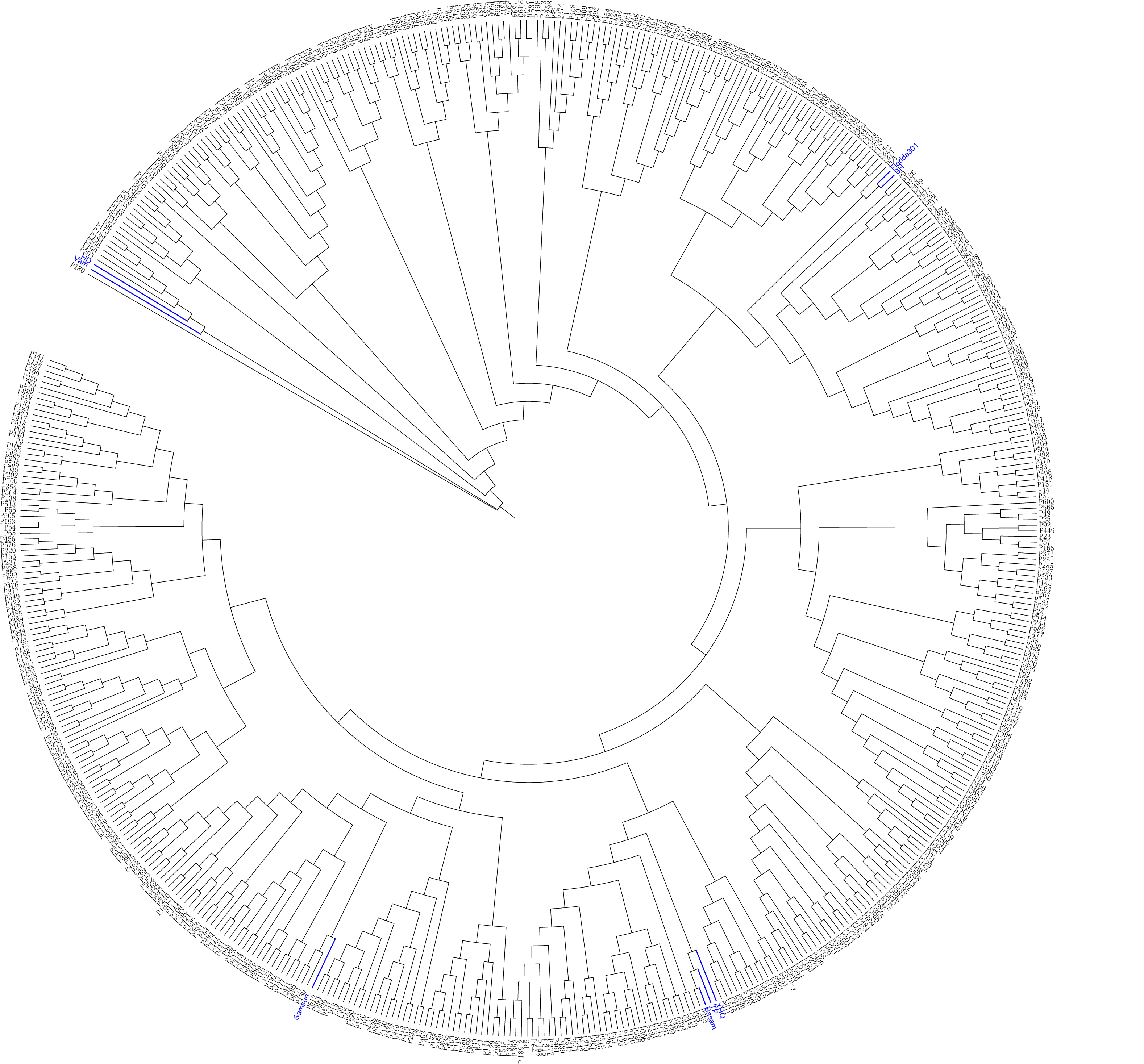

Supplement: Supplementary file 8 [file Image_8.TIF]
