# Supplementary material for: Identification of QTLs Associated With Agronomic Traits in Tobacco via a Biparental Population and an Eight-Way MAGIC Population
Source: Front Plant Sci. 2022 Jun 6;13:878267. doi: 10.3389/fpls.2022.878267 (PMC9207565; doi:10.3389/fpls.2022.878267)
Supplement: Supplementary Table 2 — The statistics of the high-density single nucleotide polymorphism-based genetic maps in the BH/XHJ population from the 430K SNP genotyping assay. [file Table_2.DOCX]

**Supplementary Table 2** The statistics of the high-density single nucleotide polymorphism–based genetic maps in the BH/XHJ population from the 430K SNP genotyping assay

| Linkage group | Total markers | Linkage group length (cM) | Average marker distance (cM) |
| --- | --- | --- | --- |
| 1 | 252 | 255.58 | 1.01 |
| 2 | 221 | 198.63 | 0.90 |
| 3 | 171 | 162.09 | 0.95 |
| 4 | 197 | 177.38 | 0.90 |
| 5 | 207 | 176.91 | 0.85 |
| 6 | 135 | 174.12 | 1.29 |
| 7 | 172 | 148.43 | 0.86 |
| 8 | 181 | 172.84 | 0.95 |
| 9 | 183 | 176.45 | 0.96 |
| 10 | 153 | 160.98 | 1.05 |
| 11 | 147 | 140.24 | 0.95 |
| 12 | 230 | 218.48 | 0.95 |
| 13 | 204 | 194.47 | 0.95 |
| 14 | 123 | 100.09 | 0.81 |
| 15 | 171 | 150.45 | 0.88 |
| 16 | 128 | 130.21 | 1.02 |
| 17 | 143 | 143.10 | 1.00 |
| 18 | 136 | 182.80 | 1.34 |
| 19 | 120 | 111.41 | 0.93 |
| 20 | 187 | 190.44 | 1.02 |
| 21 | 127 | 135.38 | 1.07 |
| 22 | 112 | 148.46 | 1.33 |
| 23 | 91 | 120.00 | 1.32 |
| 24 | 143 | 151.49 | 1.06 |
| Total | 3934 | 3920.43 |  |
